# Staging of lung cancer in a tertiary care setting in Sri Lanka, using TNM 7^th ^edition. A comparison against TNM6

**DOI:** 10.1186/1756-0500-5-143

**Published:** 2012-03-14

**Authors:** Dinesh LB Dassanayake, Thushara M Muthunayake, Kapila HMP Senevirathna, Anoma Siribaddana

**Affiliations:** 1 Respiratory Unit, Teaching Hospital Kandy, Kandy, Sri Lanka; 2 Department of Radiology, Teaching Hospital Kandy, Kandy, Sri Lanka

**Keywords:** Lung, Cancer, Staging, TNM7

## Abstract

**Background:**

Lung cancer is a leading cause of cancer-related mortality in Sri Lanka and throughout the world. The latest staging system for lung cancer is the tumor node metastasis (TNM) 7^th ^edition in which there are major changes to the previous version. The objective of our study was to find out the implications of TNM7^th ^edition on lung cancer staging in a resource limited setting, and to compare it with the previous TNM 6^th ^edition.

**Methods:**

Patients with histologically proven lung cancer consecutively presented to respiratory unit of Teaching Hospital Kandy, Sri Lanka were recruited to the study over a period of one year from April 2010 to March 2011. They were staged using CT, ultrasound scan of abdomen, bronchoscopy and CT spine and brain when necessary. Staging was done using TNM 7 as well as TNM6. Surgical or non-surgical treatment arms were decided on staging and the number of patients in each treatment arm was compared between the two staging systems.

**Results:**

Out of 62 patients, thirty four patients (54%) had metastatic disease and 19 (30%) of them had pleural effusions (M1a), while 15 (24%) had distant metastasis (M1b). When compared to TNM6 there was no difference in the number of patients in T1 category, but the number in T2 was higher in TNM7 (25 Vs 20). Similarly the number in T3 group was higher in TNM7 (11 Vs 5) and the number in M category was doubled (34 Vs 17 [Chi-6.46, *p *= 0.011]) compared to TNM 6. The number of patients suitable for surgery were 17(27.5%) in TNM 7 and 18(29%) [Chi-0.02, *p *= 0.88] in TNM6.

**Conclusions:**

This study shows that a significant proportion of patients were having advanced disease with distant metastasis on presentation. The number of patients falling to stage IV is significantly higher when staged with TNM7 but there was no significant difference in the number of patients undergoing surgery when TNM 7 was used compared to TNM6.

## Background

Lung cancer is a leading cause of cancer-related mortality in Sri Lanka and throughout the world. Accurate staging of lung cancer is very important to offer appropriate treatment [1]. Latest staging system for lung cancer is the tumor node metastasis (TNM) 7^th ^edition published by the International Union Against Cancer (UICC) and American Joint Committee on Cancer using the data base of International Association for the Study of Lung Cancer (IASLC) in December 2009 [2]. This new edition formulated on data from 68463 patients from 20 countries spanning Asia, Australia, Europe and North America, is based on survival data and therefore reflects the prognosis better than the previous 6^th ^edition of TNM [2,3]. One of the changes in the TNM 7^th ^edition is the classification of pleural effusion as a metastasis (M1a) descriptor, which was classified as a T4 descriptor in the previous version [3]. This shift could result in a major change in group staging as it would group these patients to stage four. As a significant proportion of patients have pleural effusions it is expected that the patients falling in to stage four will go up with the latest staging system.

Assigning patients to surgical or nonsurgical treatment arms are decided by several criteria, and the stage of the cancer is an important criterion in deciding the treatment option. Therefore changes in the new classification and staging would alter the number of patients undergoing surgical treatment as well as non-surgical treatment. This will have a great clinical effect in the management of patients with lung cancer. However accurate lung cancer staging in a resource limited setting like ours will be difficult due to lack of facilities like PET/CT, MRI and mediastinoscopy.

The objective of our study was to find out the implications of TNM7^th ^edition on lung cancer staging in a resource limited setting, and to compare it with the previous TNM 6^th ^edition.

## Results

There were 62 patients with histologically confirmed lung carcinoma. Out of them 50 (81%) were males and 12 (19%) were females. Forty two (84%) males and 1 (8%) female were smokers [Chi-113.1,*p *< 0.0001]. Mean age of males was 61 (range 34 to 84) and females was 57 (range 38 to 79) years. Four (8.0%) out of 50 males and 5 (41.6%) out of 12 females were below the age of 50 [Chi-6.33, *p *= 0.011].

**Figure 1:**
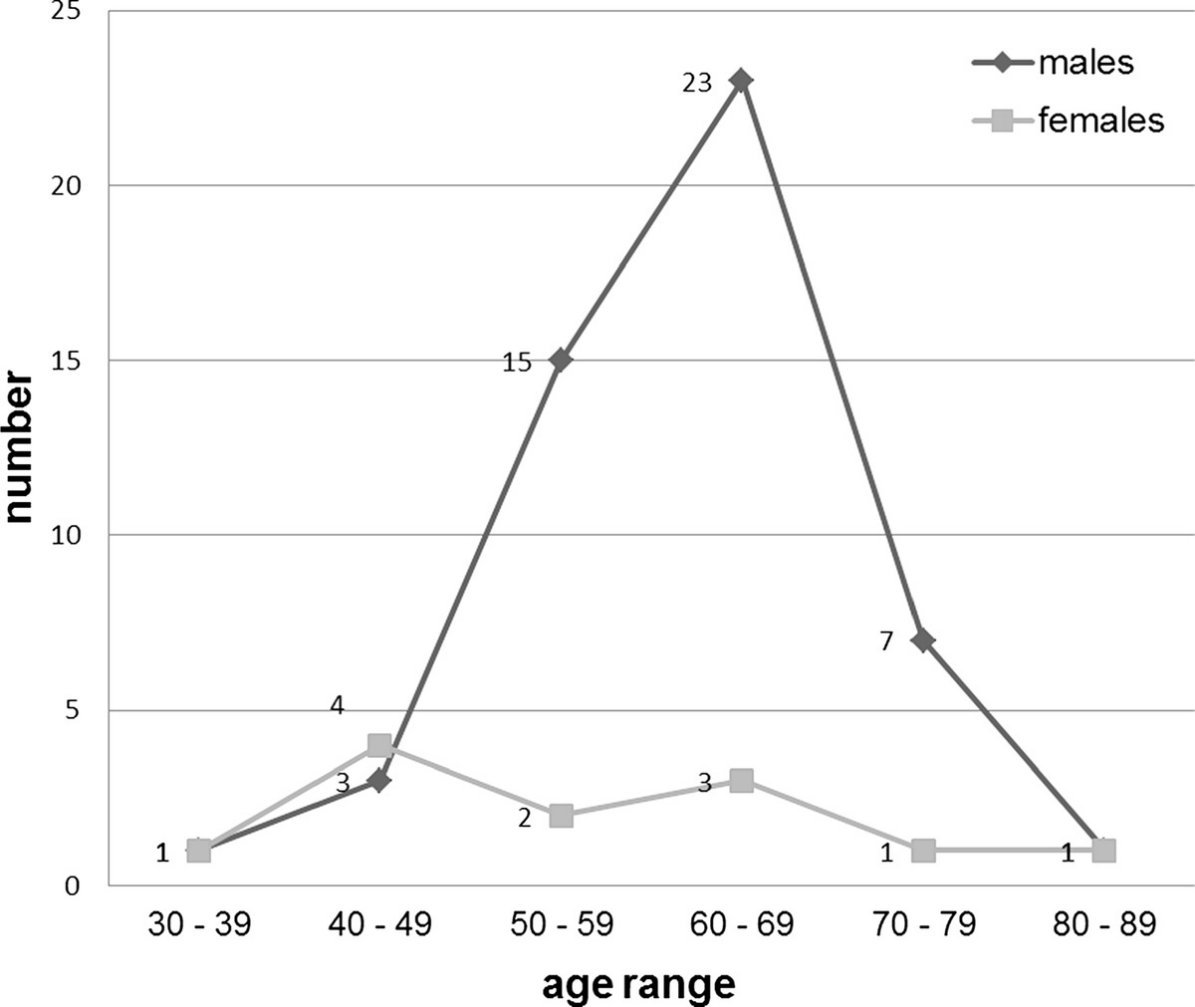
**Age specific incidence of lung cancer in males and females**.

Age specific incidence of lung cancer in males and females is shown in Figure 1.

The commenest histological type was squamous cell carcinoma reaching 36 (58%) (males 32, females 4). Fifteen (24%)(males 9, females 6) were having adenocarcinoma,5 (8%) small cell carcinomas(all were males) and 6 (9%) (males 4, females 2) patients were having other types of carcinomas.

Chest imaging revealed bilateral lesions in 4 (6.5%) patients while 30 (48.4%) had right sided lesions and 28(45.1%) had left sided lesions. Table 1 shows the distribution of lesions on chest imaging.

**Table 1:** Distribution of lesions on chest imaging

Site of lesion	Total*	hilar	Upper lobe	Middle lobe /Lingula	Lower lobe	Pleural effusion
Right	30	7	12	3	3	10

left	28	10	7	1	6	9

*Four patients had bilateral lesions

Masses, effusions, collapsed areas, multiple nodules and cavities were the types of lesions found on chest imaging. Some patients had a combination of the lesions. Twenty five patients (42%) had a lung mass on imaging, 10 (17.7%) had a mass with effusion, and 9(16%) had a mass with collapse of a lobe. Six (13%) presented with effusion only while 3 (6.3%) had multiple nodules. Collapse of a lobe was the finding in 2 (3.2%) and 1 patient (1.6%) had a cavity on chest imaging.

**Figure 2:**
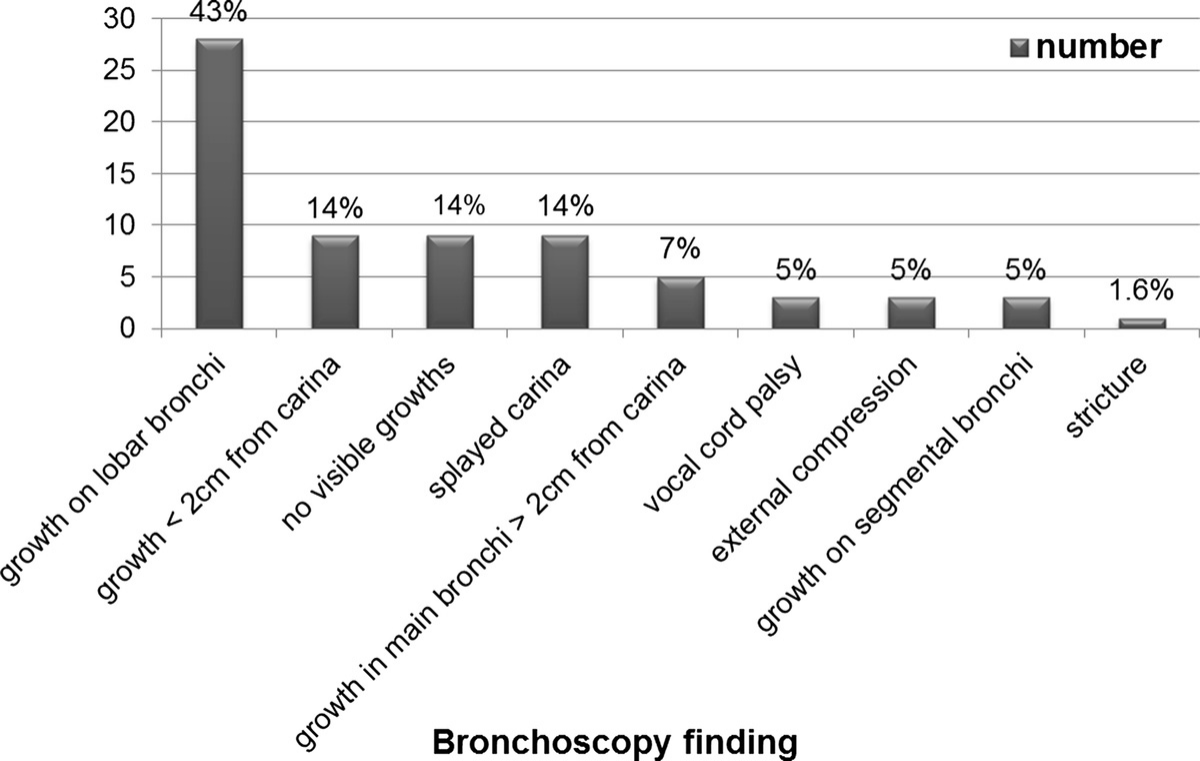
**Bronchoscopic appearance in patients with lung cancer**.

The commenest bronchoscopic lesion was the presence of a growth on a lobar bronchus (43%). Figure 2 shows the bronchoscopic appearance in patients with lung cancer.

Thirty four patients (54%) had metastatic disease on admission. Out of them 19 (30%) had pleural effusions (M1a), while 15 (24%) had distant metastasis (M1b). Out of distant metastasis 7(47%) had multiple metastasis. Three (20%) had liver metastasis, 2 (13%) adrenal, 2 (13%) brain and 1(7%) had bone metastasis.

TNM staging was done using TNM 7^th ^as well as TNM 6^th ^and the number of patients in each category is shown in Table 2.

**Table 2:** TNM staging

TNM stage	TNM 7	TNM 6
T1	3	3

T2	25	20

T3	11	5

T4	23	34 (T4 by pleural effusions-19)

M	34 (pleural effusions M1a-19)	17

The number of patients in T3 group was higher in TNM7 (11 Vs 5) and the number in M category was doubled (34 Vs 17, [Chi-6.46, *p *= 0.011]) compared to TNM 6.

Group staging according to each TNM system is shown in Table 3.

**Table 3:** Group staging according to each TNM system

Stage	TNM 7	TNM 6
IA	2	2

IB	3	3

IIA	0	1

IIB	3	3

IIIA	9	9

IIIB	10	25

IV	35	19

The number of patients in stage IIIB were lower in TNM7 (10 Vs 25) while the number in stage IV was higher (35 Vs 19) [Chi-9.75, p0.002].

When TNM 7^th ^was compared with TNM 6^th^, 21(33.8%) patients had a change in the TNM classification while 20 (32.2%) patients had a change in the group staging. Out of them 17(27.5%) patients were placed to a more advanced stage and 3 (4.8%) patients were staged to a lesser advanced stage by the new system.

Management plan according to the stage is shown in Table 4.

**Table 4:** Management according to each TNM system

Management plan	TNM 7		TNM 6	
	
	number	%	number	%
surgical	17	27.5	18	29

non-surgical	45	72.5	44	71

The number of patients suitable for surgery were 17(27.5%) in TNM 7 and 18(29%) [Chi-0.02, *p *= 0.88] in TNM6.

## Discussion

As with previous similar studies [1] this study confirms the male predominance in lung cancer incidence which could be due to high prevalence of active smoking among males. According to Cancer Registry of Sri Lanka, adenocarcinomas are commoner 69% than squamous carcinomas 28% [4]. However in our series squamous carcinomas were common 58% and adenocarcinoma contributed only to 24% (Chi-29.54, *p *< 0.0001). The discrepancy could be due to the small sample size in this series. Not all patients are referred to respiratory unit for diagnosis, and some patients are diagnosed in other units like medical, surgical and thoracic surgical unit therefore causing a selection bias which could be another reason for the discrepancy. However this could not have caused a significant impact on staging as, apart from small cell carcinomas which are aggressive other non-small cell carcinomas behave similarly.

An interesting finding is that the age specific incidence between males and females differ significantly. In males the peak incidence was between 60 to 69 age group, which was parallel to the national incidence data. The highest number in female group was between 40 to 49 age group. According to national data, peak incidence in females occurred in 50 to 54 age group. The proportion of patients below 50 years of age is higher in females. Compared to UK [5], females with lung cancer in Sri Lanka were younger according to national statistics, which is further strengthened by our data. Whether this is connected with the exposure to fire wood smoke during cooking, needs to be investigated.

The presence of distant metastasis at presentation was higher (24%) compared to the western figures (21%) [6]. This could have been due to the late presentation of patients in our country. The number of patients who had brain secondaries (13%) was less compared to the study done by Leslei et al (19%) but presence of multiple secondaries (47%) was higher compared to the same study (33%) [6].

Usually about 15% of patients with lung cancer will have pleural effusions [7], but in our study it was very high reaching 56% [Chi-34.9,*p *< 0.001]. This was a major finding in our study which again provides evidence to the late presentation of patients.

When the tumors were staged using TNM 7 and TNM 6 separately it was evident that there was no difference in the number of patients in T1 category, but the number in T3 group was higher in TNM7 (11) than with TNM 6 (5). This is due to the reclassification of tumors larger than 7 cm as T3 and change in classification of separate tumor nodule(s) in the same lobe from T4 descriptor to T3 in the recent staging system (3). Reclassification of pleural effusions as M1a has several impacts on the T descriptor in new system. Tumors that could be classified as T2 according to size fell into T4 group in TNM6. In new system they were reclassified as T2M1a. Similarly tumors that fit into T3 by size fell into T4 in TNM6 due to pleural effusions and they were reclassified as T3M1a. This change was another reason for increase in number of patients in T2 and T3 categories in TNM 7. When compared to the study done by Carvalo L et al, the number of patients with a change in staging with TNM 7 was higher (32% Vs 25.1%) [8].

The most important finding in our study was the number of patients in M category being doubled (34) compared to the old TNM 6^th ^edition (17). This is due to the reclassification of pleural effusion as M1a in TNM7. In TNM 6 it was classified as T4. Due to the same change the number of patients in Stage IV disease in TNM 7 has increased and the number in stage IIIB has decreased compared to TNM 6. Therefore the number of patients falling in to the category of "poor prognosis" will be increased when TNM 7 is applied for staging.

However when the two TNM systems were compared there was only 1.5% reduction in the number of patients undergoing surgery when TNM 7 was used compared to TNM 6 which was not statistically significant. This is because the patients with pleural effusions are considered inoperable in both the staging systems.

## Conclusions

This study shows that a significant proportion of patients with lung cancer were having advanced disease with distant metastasis on presentation. The number of patients falling to stage IV is significantly higher when staged with TNM7 but there was no significant difference in the number of patients undergoing surgery when TNM 7 was used compared to TNM6.

### Availability of supporting data

The data sets supporting the results of this article are included within the article.

## Methods

The study was carried out in the Respiratory Unit of the Teaching Hospital Kandy from 30^th ^April 2010 to 31^st ^March 2011. The teaching hospital Kandy is the second largest hospital in Sri Lanka and the respiratory unit is the major referral center for chest diseases for nearly one third of the island. The ethical clearance for the study was granted by the ethical committee of the Teaching Hospital Kandy. Written informed consent was obtained from participants.

Patients referred to the Respiratory Unit with suspected lung cancer were screened clinically and were subjected to chest radiography. Those who had radiographic features suggestive of lung cancer underwent fiber optic bronchoscopy with bronchial biopsy, bronchial brushings and bronchial washings for cytology. Those patients with lesions placed peripherally underwent ultrasound guided or CT guided biopsy of the lesions. Patients who had pleural effusions underwent pleural biopsy and aspiration cytology in addition to bronchoscopy. All those patients who were confirmed as having lung cancer on histology were recruited to the study and were staged according to TNM7 as well as TNM6 for comparison. All patients underwent CT thorax and upper abdomen which was the main tool used for staging. Bronchoscopy was also used as a tool for staging. Ultrasound abdomen and chest, × ray spine, serum calcium, serum alkaline phosphatase, CT brain and spine were used in some patients to detect secondaries if they had clinical features of direct spread or dissemination to distant organs. TNM 7^th ^edition was used to decide whether the patient should undergo surgical or non-surgical management. Up to group stage IIIA was considered as suitable for surgery and others were considered for non-surgical management. The patients were also staged using the TNM 6^th ^edition for comparison.

Clinical data were collected using a symptom check list and an interweaver administered questionnaire. Investigation details such as bronchoscopy and CT findings together with the clinical findings were entered into a data sheet.

Data were analyzed for descriptive statistics using percentages, and the TNM 7^th ^edition was compared against TNM 6^th ^edition using Chi-square statistics.

## Competing interests

The authors declare that they have no competing interests.

## Authors' contributions

DLBD was involved in designing, acquisition of data and drafting the manuscript of the study. MTM was involved in statistical analysis, interpretation of data and review of the manuscript. HMKPS was involved in the acquisition of data, and designing the study. AS conceived the study, reviewed the manuscript and approved the final manuscript. All authors approved the final manuscript.

## Authors' information

DLBD is the corresponding author and is a Senior Registrar in Respiratory Medicine attached to the Respiratory Unit of the Teaching Hospital Kandy. He has MBBS and MD Medicine. He is also a member of the Collage of Physicians Sri Lanka.

MTM is a Senior Registrar in Radiology attached to the Department of Radiology of the Teaching Hospital Kandy. He has MBBS and MD Radiology.

HMKPS is a medical officer attached to the Respiratory Unit of the Teaching Hospital Kandy. He has MBBS and is enrolled in the Diploma of Tuberculosis and Chest Diseases.

AS is the Consultant Chest Physician in charge of the Respiratory Unit of the Teaching Hospital Kandy. She has MBBS, MD Medicine and MRCP degrees. She is also a member of the Collage of Physicians Sri Lanka.
